# Medium-term outcome of the Libra^®^ cemented versus cementless stems in primary dual mobility total hip arthroplasty

**DOI:** 10.1186/s12891-023-06799-8

**Published:** 2023-08-21

**Authors:** Ayman Ebied, Ahmed Ali Ebied, Ismail Badr, Mostafa Affara, Sameh Marei

**Affiliations:** Department of Orthopedic Surgery, Faculty of Medicine, Menoufia University, Shibin el Kom, Menoufia 32511 Egypt

**Keywords:** Total hip arthroplasty, Dual mobility, libra^®^, Cemented, Cementless

## Abstract

**Introduction:**

Despite the increasing use of cementless stems in total hip arthroplasty, the cemented stem has played a valuable role in the armamentarium of orthopedic surgeons. This study aims to compare two types of Libra® stems SERF, one cemented (Libra® C) and the other cementless hydroxyapatite coated (Libra® HA) that were conducted to analyze the medium-term outcome regarding their behavior and longevity.

**Methods:**

This is a retrospective study for patients who received primary total hip arthroplasty with Dual Mobility (DM) articulation in the period between January 2014 to January 2020 with a minimum of two years follow-up. Two-hundred hips have been identified in 196 patients. One hundred forty-three Libra® cementless versus fifty-seven Libra cemented stems were implanted and the outcome of these stems is reported. All procedures were performed through the posterior approach and cemented stems were selected for elderly patients with wide medullary canals Dorr Type C. The indications for the index procedure were fractures, avascular necrosis, rheumatoid, and osteoarthritis. One hundred thirty-nine cementless DM cups were used while sixty-one hips had cemented Novae stick cups. Radiological evaluation for cup and stem positions, cement mantle, and radiolucent lines was performed, besides clinical function using the Harris Hip Score.

**Results:**

The average age of patients was 60 ± 14.8. At the latest review, none of the cemented stems was revised or awaiting revision. One cementless stem was revised because of cortical perforation. Five intraoperative fractures were observed in the cementless group, but none of them needed revision or affected the stem stability. Readmission to theatre occurred in four patients to evacuate hematoma in two, a reduction of dislocation in one, and grafting bone lysis in one.

**Conclusion:**

Cemented stems have an important role in osteoporotic patients with wide medullary canals with excellent outcomes and minimal risk of fracture.

**Level of evidence:**

Level IV.

## Introduction

Since introducing Total Hip Arthroplasty (THA) in the 1960s, the main goals were to improve bearing surfaces and implant fixation that provide implant long-term survival. A significant evolution in design, bearing surfaces, and methods of fixation was achieved in the last six decades [1, 2].

Cemented THA was considered by many surgeons across the globe as the gold standard. However, a gradual shift from cemented to cementless stems has been seen. The National Joint Registry NJR (of England and Wales) data defined cementless THA as the most implanted prosthesis in the last 15 years [3].

For the younger patient, cementless fixation of the hip implants is the standard practice with marked improvement in the designs and implant coating that enhances the potential for biological fixation and bone ingrowth to the implant surface [1, 3–5].

Double tapered, fully hydroxyapatite (HA) coated stems showed excellent initial stability and long-term survival reached 96.8% at 20 years [6]. The preference for fully versus proximally coated stems is still debatable [7]. Some argue anatomic proximally coated stems may produce less stress shielding and thigh pain when compared to straight fully coated stems [8]. The clinical significance of this argument in the long term has not been established in a randomized trial.

It remains that cemented stems have a valuable role, particularly for elderly patients with wide medullary canals (Dorr type C) and bone osteoporosis [9]. The risk of intraoperative mortality due to cement use has recently been doubted and disproved [8]. Hence, the technique and use of cemented stems should not be forgotten.

This study aims to compare two types of Libra^®^ stems SERF, one cemented (Libra® C) and the other cementless hydroxyapatite (HA) coated (Libra® HA) that was conducted to analyze the medium-term outcome regarding their behavior and longevity.

## Patients and methods

### Study design

This is a retrospective case series performed on patients who received dual mobility total hip replacement between 2014 and 2020, with a minimum follow-up of two years. Data were collected prospectively in a local database to ensure high-quality recording. A comparative study between two types of stems, one was cemented (Libra® C) and the other cementless (Libra® HA) has been performed to analyze the outcome regarding their behavior and longevity. Of one hundred ninety-six patients (two hundred hips), one hundred forty-three hips received cementless (Libra® HA) stems, while the other hips fifty-seven hips received cemented (Libra® C) stems.

### Patients’ selection

Inclusion criteria were patients who received dual mobility articulation with conventional primary femoral stem (Libra® stems) during the study period regardless of their age, original pathology, BMI, or level of activity. Exclusion criteria were any revision of femoral component or complex primary who received DM articulation with different stem types, patients in whom the stem had been inserted as part of a revision procedure, or patients with a follow-up of fewer than two years.

Patients who died or lost follow-up were not included in the analysis. Written informed consent was signed by all patients for the use of their data and images for research and publishing purposes, this is in addition to ethical committee approval from the institutional review board(1/2022ortho4-4).

### Implant design

**Fig. 1:**
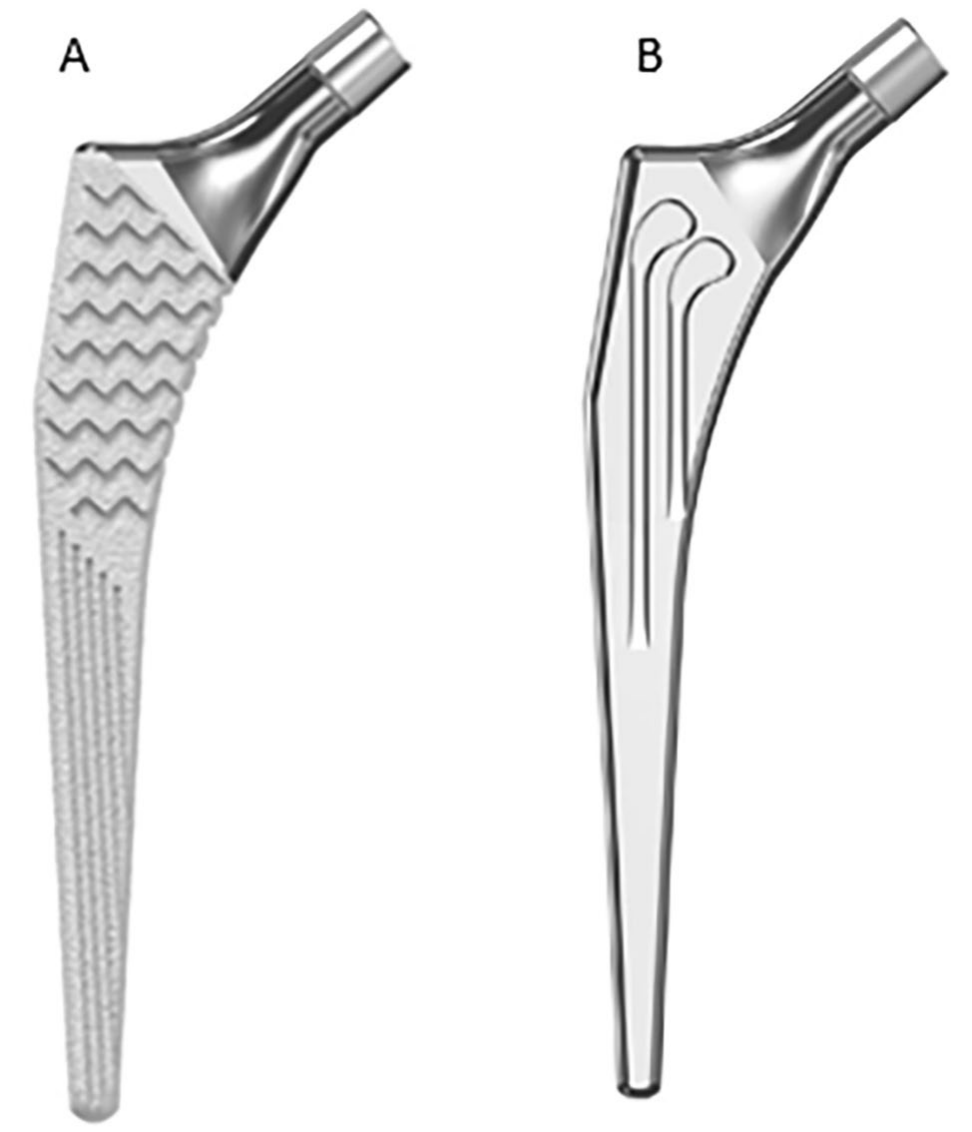
(**A**) Libra® standard offset uncemented stem (**B**) Libra® standard offset cemented stem

Libra® stems (Serf, Décines-Charpieu, France) are straight collarless self-locking stems dedicated to primary and revision total hip arthroplasty. They are characterized by their long, double-taper geometry. The stem has an 11/13 taper which needs to be combined with matching heads taper. Cementless stems are made of titanium alloy and hydroxyapatite-coated (Fig. 1A), while cemented stems are fully polished high-nitrogen stainless steel (Fig. 1B) [10].

### Surgical technique

The procedures were performed through the posterior approach. and acetabular preparation, acetabular reaming, and cup positioning for all hips were performed. First, osteophyte removal, if any to visualize the acetabular contour, true floor, and the crucial landmark, transverse acetabular ligament (TAL). Then progressive reaming of the acetabulum exposes bleeding subchondral bone and obtains adequate stability of the reamer. A line-to-line trial cup was then inserted to verify the primary stability of the cup, cup size, and orientation with cementless cups. The final cementless cups were inserted parallel to the transverse acetabular ligament.

On the femoral side, the neck cut was inclined 45 degrees with the intramedullary axis. Box osteotome was used to open the medullary canal at the Pyriformis fossa. A polished canal finder was chosen to define the medullary canal direction yet preserve the cancellous bone. Sequential broaching of the proximal femur was performed till vertical and rotational stability were obtained. Cemented Libra® stems were employed in elderly patients with Dorr [9](type C medullary canals).

Cementless Libra® stems were selected for patients with good bone quality Dorr types A and B medullary canals. The size of the stem is determined by the biggest broach that achieved vertical and rotational stability. A Cementless stem the same size as the last broach was inserted by hand pressure and hammer impaction till the stem is fully seated.

Cemented Libra® stems were chosen when Dorr Type C medullary canals were encountered, and bone osteoporosis was clear. Canal preparation was performed using sequential sizes of the broaches. Removal of loose cancellous bone while maintaining the strong cancellous trabecular bone was aimed. This was followed by plug insertion 5 mm distal to the tip of the stem, irrigation using saline, and drying of the medullary canal using sterile gauze and a catheter connected to suction.

Retrograde filling of the medullary canal by standard-setting antibiotic-loaded bone cement using a syringe gun was performed. Then cement pressurization within the medullary canal using the medullary top seal and hand pressure was performed before stem insertion using hand-sustained pressure to the desired level.

### Patient evaluation& Follow‑up

Follow-up visits at 6 weeks, 3 months, 6 months, 1 year, then yearly, in which complete functional evaluation was done. Radiological evaluation through anteroposterior and lateral radiographs of the operated hip. Clinical evaluation of all patients using the modified Harris Hip Score (mHHS) with a minimum follow-up of 2 years [11].

The following data were collected and analyzed including implant details, postoperative complications, or revisions, loosening of implant whether cement or cementless, radiolucent lines in any of Gruen zones, stem subsidence, infection, radiographic evidence of bone ingrowth around cementless stems. The endpoint for this study was stem revision for any reason.

### Statistical analysis

IBM SPSS version 25.0 (SPSS Inc., Armonk, NY) was used for the statistical analysis of data. Categorical variables were compared using the chi-square or Fisher’s exact tests, while continuous variables were compared using the student’s t-test or Mann-Whitney U test, and the mean and standard deviation (SD) were calculated for descriptive variables. Statistical significance was set at a P-value < 0.05.

## Results

### Patients’ characteristics

This case series comprised ninety-nine males (102 hips) and 97 females (98 hips), 110 hips were right, and 90 were left. The patient’s age at the index procedure was 59.9 ± 14.8 years (range, 18 to 85). One hundred forty-three hips received DM articulation over cementless stem (Libra® HA) (71.5%), while fifty-seven hips received DM articulation over cemented stem (Libra® C) (28.5%). The indication for THR was femoral neck fracture for 87 hips (43.5%), avascular necrosis for 51hips (25.5%), failed fixation of femoral neck fractures and trochanteric fractures in 21 hips (10.5%), primary osteoarthritis for 19 hips (9.5%), Primary acetabular fracture or failed fixation for 10 hips (5%), osteoarthritis secondary to congenital hip dysplasia for 6 hips (3%), osteoarthritis secondary for Perthes disease for four hips (2%), and post septic osteoarthritis for two hips (1%). (Table 1)

**Table 1:** The characteristics of all patients and both stem groups

			Libra® C (n = 57 hips)	Libra® HA (n = 143 hips)	Total (n = 200 hips)
HHS			90.56 ± 4.9	92.7 ± 5.7	92.1 ± 5.5
Follow up (Months)			45.2 ± 15.8	43.9 ± 13.8	44.3 ± 14.4
Age (mean ± SD)			71 ± 7.4	55.4 ± 14.6	59.9 ± 14.8
Cup size in mm (mean ± SD)			49.6 ± 4	54 ± 4.4	52.8 ± 4.7
stem size in mm (mean ± SD)			4.6 ± 1.5	5.1 ± 1.7	5 ± 1.7
Gender		Male	25	77	102
		Female	32	66	98
Side	Right		33	77	110
	left		24	66	90
Indication of operation	Post septic hip arthritis with hip ankylosis		0	2	2
	AVN		10	41	51
	Dysplastic Hip		1	5	6
	Failed #Trochanteric		1	8	9
	Failed screw fixation NOF#		3	9	12
	Neglected Perthes Disease		0	4	4
	NOF#		34	53	87
	OA		7	12	19
	Primary acetabular fracture or failed fixation		1	9	10
Cup type	Novae® E TH		4	103	107
	Novae® Sunfit TH		1	9	10
	Novae® Coptos TH		0	10	10
	Others cementless design		3	8	11
	Novae Stick		49	13	62

### Patient information

Cementless Acetabular component was used in138 hips (Novae® E TH cup in 107 hips, Novae® Sunfit TH in10 hips, Novae® Coptos TH cup in ten hips, others 11), while sixty-two hips received cemented acetabular component; Novae® Stick (3 was combined with Kerboull cross ring). Twenty-one hips were hybrid or reverse hybrid. the mean cup size was 52.8 ± 4.7 mm (range 43 to65 mm), while the mean stem size was 5 ± 2 (mean +/- SD) (range 1 to 10). (Table 1)

### Comparison of clinical outcomes between both groups

No statistically significant differences between patients’ populations in both stem groups regarding gender (p = 0.214), age(p = 0.639), or primary indication for arthroplasty (p = 0.508). However, the associated comorbidity was higher in the cemented than in the cementless group. The ASA grade of patients was on average higher in the cemented group (ASA III & IV) than the cementless group who were more fit and active preoperatively.

Eight patients from the cemented group Libra ®C were dead at the latest review. The cause of death was unrelated to the surgical intervention in all patients. The time of death from index surgery was > 24 months from surgery in four and they were not excluded from the analysis. While patients who died in the first-year post-surgery were excluded. The mean follow-up period was 44.3 ± 14.4 months (range 24 to 75), and the mean mHHS was 92.1 ± 5.5(range 60 to 100).

### Radiographic assessment

**Fig. 2:**
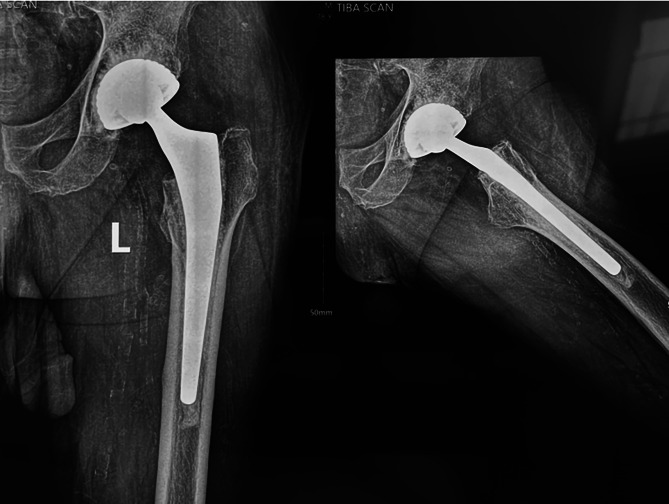
Fully cemented Dual Mobility THA in a 73-year-old male that was performed 4 years before for fracture NOF that shows stable cement mantle with no evidence of loosening on the femoral or acetabular sides

Regarding the cemented group, none of the stems was revised at the time of the latest review or awaiting revision (Fig. 2). Five stems had non-progressive radiolucent lines one mm at the bone cement interface in Gruen zones 3 or 5, but the stems were well fixed and stable.

**Fig. 3:**
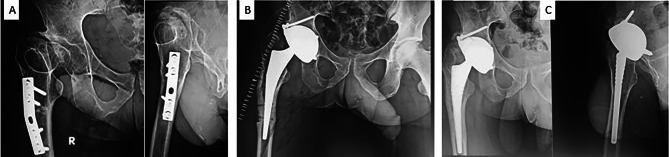
32-year-old male patient had THA for secondary arthritis post-Perthes’ disease. (**A**) preoperative x-ray that shows the proximal femoral deformity and plate used for varus osteotomy in his childhood (**B**) immediate post-operative x-ray with Libra cementless stem inserted through the site of previous deformity and screw holes evident around distal part of the stem. (**C**) 4 years post-operative with stem integration and filling of bone defects

**Fig. 4:**
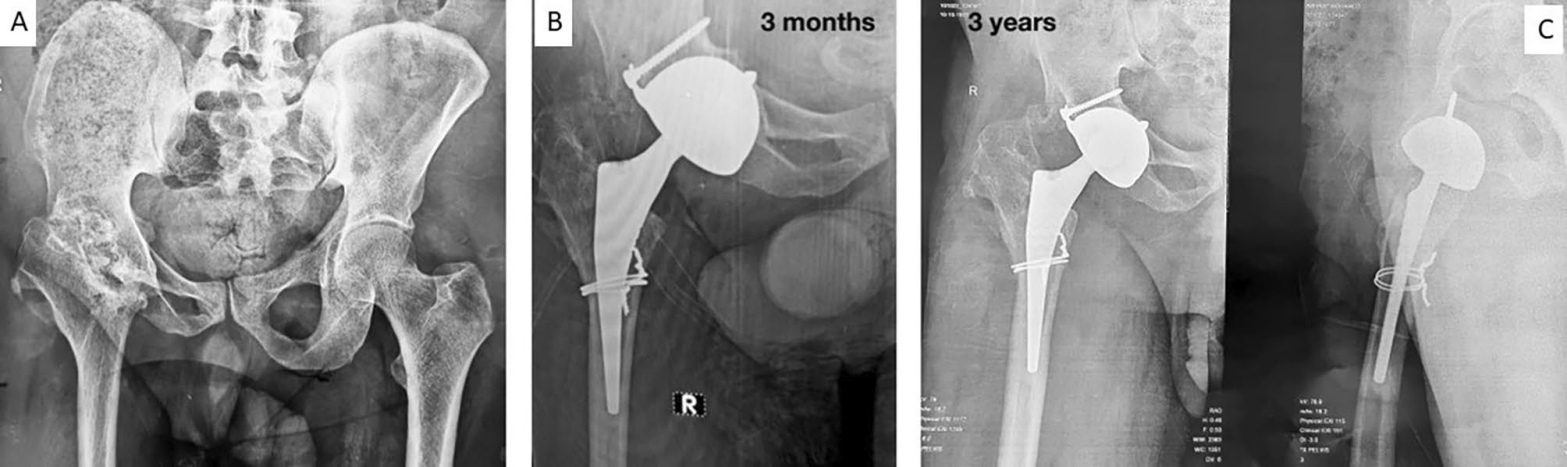
53 years old male patients who had hip arthritis secondary to septic arthritis in childhood and pelvic support osteotomy (**A**) preoperative x-ray with advanced arthritis limb length discrepancy and valgus position of the proximal femur (**B**) Libra cementless stem and Novae® E TH DM cup, intraoperative crack of the proximal femur during stem insertion was observed and protected with wires (**C**) 3 years post-operative with full bone ingrowth around the stem

All cementless stems were stable with bone ingrowth at the latest review and only one stem was revised within a week from primary surgery because of stem perforation which was a technical error at the time of insertion. The stem was replaced by a Sagitta EVL R cementless stem. All other cementless stems were not revised or awaiting revision including those that were used in complex cases with femoral deformities (Figs. 3a-b and 4a-b). The Libra® cementless stem with a porous and fully HA-coated surface was observed to enhance bone ingrowth (Figs. 3c and 4c).

The Libra cemented stem was chosen for patients with wider medullary canals type C in Dorr’s classification. This may give the impression that this group of patients was older and had a higher incidence of comorbidities. However, reviewing the data set no statistical difference was found in the ASA grade of patients between patients who received cemented versus cementless stems.

### Patient readmission

Four patients were readmitted to the theatre for other reasons. Two of these readmissions were within the first three weeks to evacuate hematomas that developed with continued wound drainage and were attributed to the use of new oral anticoagulants (NOAC). The third patient was readmitted to the theatre for closed reduction of hip dislocation and later for cup exchange to a constrained liner.

**Fig. 5:**
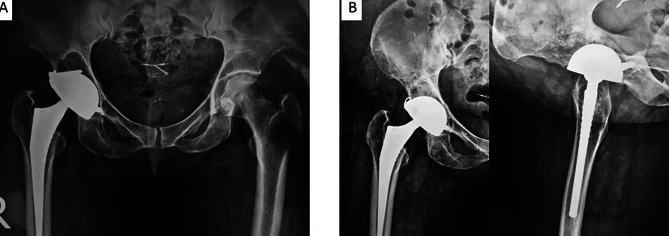
46 years female patient who had THA with cementless stem and Novae® E TH cup (**A**) 3 years post-operative started to develop cysts around the cup flange (**B**) 1 year after the bone graft using the trap door technique

The last patient had developed endosteal cavitation around a Novae® E TH cup with superior bone resorption (Fig. 5A). The cup was however stable, and an impaction graft of fresh frozen allograft was performed by the trap door technique (Fig. 5B).

### Complications

There were four intraoperative complications related to the cementless femoral stems in the form of proximal femoral calcar split that did not affect the stability of the implant and were protected with cerclage wiring and delayed return to full weight bearing (Fig. 4). Three patients in the cementless group had limb length discrepancy of 10 mm that was accepted by the patients and corrected by shoe raise.

The survival of cemented stems was 100% while the survival of the cementless stem was 99.3%.

## Discussion

The most important finding in this study is that cemented stems have achieved similar excellent results to cementless ones with less incidence of postoperative complications in the early and medium term.

The comparison in this study was between two types of stems. The cementless one is a double tapered fully HA coated which has previously shown highly successful outcomes by different manufacturers [3]. The new report here is on the cemented version Libra® C stem. Which is again double tapered and fully polished made of N stainless steel.

Cemented stems have achieved excellent outcomes over the years with different philosophies in explaining their success. The success of the Exeter stem, for example, has been attributed to the double-tapered configuration and fully polished surface that allows controlled subsidence within the cement mantle [12]. Thus, proximal loading of the femur is achieved, and stress shielding is minimized [13]. On the contrary, broad cemented stem designs with cement mantle as thin as one mm achieved a good record of survival on what is called the French paradox [14]. Explanations for this paradox like strong cortical support for the cement mantle and stability of the implant have been suggested.

The Libra® C stem is a double-tapered stem with a polished surface and fine grooves around the proximal part. The broach system allows for 2 mm of cement mantle circumferentially. What is important yet is the cementing technique. Retrograde filling of the medullary canal and cement pressurization is mandatory to create a stable and integrated cement mantle for stem long-term stability.

It is worth noting that the cemented version of the stem was selected for patients with Dorr type C medullary canals who were treated for femoral neck fractures, OA, AVN, and failed fixation of hip fractures. Cementless stems have a high incidence of intraoperative fractures in this category of patients with wide medullary canals and osteoporosis. Using the cemented stem did not cause any intraoperative complications.

When a DM articulation is implanted attention should be paid to the stem design. A stem with a narrow-polished neck is advisable to reduce the chance of impingement between the stem and the mobile polyethylene liner which would increase PE wear particles (the third articulation) [15]. The libra® stems (both cemented and cementless) have a narrow neck that reduces the risk of creating a third interface or producing PE wear particles; when this occurs, it may affect the long-term survival of these implants. A narrow stem neck is also a favorable criterion in a DM articulation to reduce the incidence of Intra-Prosthetic Dislocation (IPD) a complication that was not observed in any of the hips in this study.

The cementless Novae® cups have been developed from the original DM design by Gilles Bousquet [16]. Cementless versions of the cups are advised to be used in all primary cases while cemented DM (Novae® Stick) are reserved for cases of acetabular deficiency in association with the Kerboull Cross ring. In this series, 62 Novae® Stick cups were cemented with or without a KE ring into the acetabulum in highly osteoporotic bone. None of these cups have been revised or proved to be loose. However, the authors cannot recommend cemented DM cups being directly inserted without a ring in primary THA before long-term results become available.

Radiolucent lines were observed around two of the Novae® E TH cups. These radiolucent lines and endosteal cavitation were observed in one cup after two years after implantation. At the time of surgical exploration, the cup was stable. However, the screw used for extra-articular fixation was found to be loose. Corrosion between the screw and flange might have contributed to the development of these supra-acetabular cysts. Impaction of fresh frozen allograft was performed through a trap door on the side of the ilium and implants were kept in position (Fig. 5). There is another case where the radiolucent line is noticed as superior to a Novae® E TH cup, but the patient is still under review.

Intraoperative fractures are not uncommon complications while using cementless stems [17]. This complication is notably observed in the elderly population with fractured neck of the femur [17]. The NICE guidelines recommend the use of cemented stems in this patient category [18]. Four patients in this series had intra-operative split around the proximal femur. Two of them happened during the broaching process and the other two during the stem insertion. The splits did not affect the stability of the stem and wires were used to safeguard against extension of the bone split. Delayed weight bearing for 6 weeks was adequate to allow for the healing of these bone cracks and stem integration within its bony bed.

## Conclusion

The Libra® C cemented stem achieved 100% survival in the medium term for patients with wide medullary canals and osteoporosis who received DM THA for fracture neck of femur, OA, and AVN. The cementless Libra® HA stem has achieved a high success rate of 99.3% in patients with good bone stock with no revision for aseptic loosening and minimal complications in the medium term. This report supports the continued use of both stems depending on the patient’s bone quality. However, long-term results should be reported.

## Data Availability

Available on request, the corresponding author is responsible for data availability (I B).

## List of Abbreviations

THA: Total Hip Arthroplasty
NJR: National Joint Registry
DM: Dual Mobility
HA: Hydroxyapatite
TAL: Transverse Acetabular Ligament
SD: Standard deviation
HHS: Haris HIP SCORE
NOAC: New oral anticoagulants
IPD: Intra-prosthetic dislocation
OA: Osteoarthritis
AVN: Avascular Necrosis
